# Ge/Al and Ge/Si_3_N_4_/Al Core/Shell Quantum Dot Lattices in Alumina: Boosting the Spectral Response by Tensile Strain

**DOI:** 10.3390/ma15186211

**Published:** 2022-09-07

**Authors:** Ivana Periša, Marija Tkalčević, Senad Isaković, Lovro Basioli, Mile Ivanda, Sigrid Bernstorff, Maja Mičetić

**Affiliations:** 1 Ruđer Bošković Institute, Bijenička cesta 54, 10000 Zagreb, Croatia; 2 Faculty of Science, University of Sarajevo, Zmaja od Bosne 33–35, 71000 Sarajevo, Bosnia and Herzegovina; 3 Elettra Sincrotrone, S.C.p.A., Strada Statale 14, km 163.5 in AREA Science Park, Basovizza, 34149 Trieste, Italy

**Keywords:** germanium quantum dots (QDs), core–shell QDs, magnetron sputtering, tensile strain, quantum efficiency, Ge direct bandgap, multiple excitons

## Abstract

We investigated the production conditions and optoelectrical properties of thin film material consisting of regularly ordered core/shell Ge/Al and Ge/Si_3_N_4_/Al quantum dots (QDs) in an alumina matrix. The materials were produced by self–assembled growth achieved by means of multilayer magnetron sputtering deposition. We demonstrated the successful fabrication of well-ordered 3D lattices of Ge/Al and Ge/Si_3_N_4_/Al core/shell quantum dots with a body-centred tetragonal arrangement within the Al_2_O_3_ matrix. The addition of shells to the Ge core enables a strong tuning of the optical and electrical properties of the material. An Al shell induces a bandgap shift toward smaller energies, and, in addition, it prevents Ge oxidation. The addition of a thin Si_3_N_4_ shell induces huge changes in the material spectral response, i.e., in the number of extracted excitons produced by a single photon. It increases both the absolute value and the width of the spectral response. For the best sample, we achieved an enhancement of over 250% of the produced number of excitons in the measured energy range. The observed changes are, as it seems, the consequence of the large tensile strain in Ge QDs which is induced by the Si_3_N_4_ shell addition and which is measured to be about 3% for the most strained QDs. The tensile strain causes activation of the direct bandgap of germanium, which has a very strong effect on the spectral response of the material.

## 1. Introduction

Semiconductor nanoparticles and quantum dots (QDs) were extensively investigated over the past decades due to the variety of their potential application. They are very applicable in numerous modern nanotechnology devices, such as in photovoltaics, photodetectors, and optoelectronics devices [1,2,3]. For this aptness, the possibility to tune the bandgap by the QD size and the generation of multiple excitons with a single photon is important [4]. QDs evince electronic and optical properties substantially different from corresponding bulk materials due to quantum confinement. Quantum confinement is present once the semiconductor is similar in size to the Bohr exciton radius. It implies that for adequately small nano-objects, the bandgap constantly increases as the object size decreases. This effect depends on the effective mass of different carriers [5]. For instance, for a tandem solar cell, this effect would be very beneficial because differently sized QDs would harvest different parts of the solar spectrum [6]. 

Silicon and germanium QDs are frequently used herein, since both types of QDs show strong confinement effects [7,8]. Ge QDs show a more prominent quantum confinement effect than Si QDs. This stems from the fact that Ge QDs have a larger excitonic Bohr radius (24 nm) than Si QDs (5 nm). In the case of Ge QDs, quantum confinement is achieved for larger QD sizes [9]. Moreover, Ge QDs have a larger exciton binding energy and a higher electron–hole mobility (smaller effective masses of the electrons and holes) than Si QDs [10]. Therefore, it can be concluded that material properties depend on the internal structure of the QDs and the matrix type. In our previous work, we investigated the formation of Ge QDs embedded in different matrices, such as amorphous Al_2_O_3_, Si_3_N_4,_ and SiC matrices [11,12]. There, it was found that the matrix strongly influences the formation of the QDs and their conductive properties. Particularly interesting are films based on Ge QDs embedded in oxide matrices, specifically in an alumina matrix [13,14]. Alumina presents a great matrix for obtaining strong quantum confinement. It is a high-bandgap (6.2 eV) isolator and a strongly non-conductive matrix, hence QDs embedded in it should be very close to each other, which is important for photovoltaic application [15]. Furthermore, while studying materials with Ge QDs in an alumina matrix, it was found that the main downside of sputtering Ge QDs in an oxide matrix is germanium oxidation. To prevent germanium oxidation, we focus on QDs with a shell [16,17]. 

Semiconductor core-, metal-shell QDs nowadays draw strong attention due to their specific properties, leading to different nanotechnology applications [18]. In this structure, an additional manipulation of the material’s properties, besides the confinement effects, is possible due to the shell properties. In our previous work, we presented the experimental realisation of Ge/Si and Ge/metal core/shell QDs in an alumina matrix [16,17,19]. We observed that the presence of a metal shell precludes the Ge core oxidation, improves the material’s absorption level, and facilitates a significant improvement of the semiconductor absorption peak [19]. Accordingly, this led to a radical change in the material’s properties and a very important photo-current generation. The material’s properties are strongly affected by band alignment, quantum confinement, and strain. The strain considerably impacts the band structure and optoelectronic properties of semiconductor epitaxial layers [20]. By applying tensile strain, a transition from an indirect to a direct band structure is made possible as the position in the energy of the different bands varies differentially as a function of the strain that can be found in the layers. A direct bandgap semiconductor is expected to exhibit efficient radiative recombination and the possibility to obtain optical gain with reasonable injected carrier densities. In the literature, a crossover from an indirect to a direct bandgap is forecasted for tensile strain values varying between 1.55% and 2% [21]. The generation of such a tensile strain can be imputed to the thermal expansion mismatch between different materials [22]. A thick germanium layer can be transformed from an indirect into a direct bandgap semiconductor by means of silicon nitride stressor layers [21,23]. Additionally, the use of silicon nitride led to the absorption of the visible frequency range enhancing an additional six times when the Ge core was replaced by a wide bandgap insulator, such as Si_3_N_4_ [18]. Finally, self-assembled growth of QDs is desirable because it ensures more uniform QD sizes and control over QD separations, which are important for the material’s electrical properties [24]. Magnetron sputtering deposition is a relatively simple and expeditious method for the preparation of well-ordered 3D lattices of nanosized particles embedded in an amorphous alumina matrix [25,26]. The size, shape, and separation between particles can be easily adjusted by changing the deposition parameters, which makes adapting different properties of the thin film possible.

Although it is well known that the shell addition to the Ge core strongly influences the material properties, the influence of silicon nitride, which is proven to act as strong stressor for the Ge layer, is completely anexplored.

In this paper, we present the fabrication of thin film materials consisting of Ge core, Al-shell, and Si_3_N_4_/Al-shell QDs embedded in an amorphous alumina (Al_2_O_3_) matrix, by self-assembled growth achieved by means of magnetron sputtering multilayer deposition. The dots are ordered in three-dimensional (3D) QD lattices with a body-centred tetragonal (BCT) structure. The influence of the addition of Al and Si_3_N_4_/Al shells to the Ge core on the materials’ structural, optical, and electrical properties is investigated, as well as the effect of the size of the Ge core. The properties of the materials with and without a Si_3_N_4_ shell are compared.

We demonstrate the strong effect of adding an Al shell on the optical properties of the material; it causes a shift of the bandgap to smaller energies, in addition to the prevention of Ge oxidation. The addition of a thin Si_3_N_4_ shell causes a radical effect on the spectral response (i.e., quantum efficiency) of the material. Quantum efficiency (i.e., the number of created excitons) increases in the absolute value and in the energy range with the high response, which is radically widened. An enhancement of more than 250% in the quantum efficiency of the material was observed. This is probably the consequence of the strain in Ge QDs that is induced by the addition of the Si_3_N_4_ shell. A strain of nearly 3% was measured after the silicon nitride shell addition. Such strain should induce a transformation of the Ge core to a direct bandgap semiconductor. The appearance of new peaks in the spectral response with the position of the Ge direct bandgap was observed. We believe that the tensile strain, induced by the addition of the Si_3_N_4_ shell, causes an activation of the Ge direct bandgap, which is responsible for the strong enhancement of the quantum efficiency.

## 2. Materials and Methods

### Sample Preparation and Characterisation

The samples were prepared by magnetron sputtering deposition using a KJLC CMS-18 system. The base pressure in the chamber was 8* 10^−6^ Pa, and the working gas (Ar) pressure was 0.46 Pa in a continuous flow. The samples were deposited as thin films at 400 °C on Si (100) and glass substrates. 

Two series of materials with core/shell QDs embedded in an amorphous Al_2_O_3_ matrix were produced. In the first series, Ge/Al core/shell QDs embedded in alumina were produced using Ge/Al/Al_2_O_3_ multilayer deposition. The Ge layer is deposited first, and germanium in that layer forms QDs during the deposition. Aluminium covers the Ge QDs and makes shells around them. This sequence is repeated 20 times. The alumina layer thicknesses are chosen to ensure the self-assembled growth regime, so a 3D lattice of the QDs is produced. In this series, we tuned the size of the Ge core by varying the Ge deposition time. In the second series, we added an additional Si_3_N_4_ layer before the Al layer, so we used a Ge/Si_3_N_4_/Al/Al_2_O_3_ deposition sequence. The Si_3_N_4_ makes a shell around the Ge QDs, similarly to Al, and after it, the Al layer covers the structure as a second shell. In this series, we also tuned the Ge core size. 

The sputtering power for Ge, Al, and Si_3_N_4_ was 25 W, and for Al_2_O_3_ it was 140 W. The deposition times were the same (40 s) for both shell materials (Al and Si_3_N_4_), and for Al_2_O_3_ it was 200 s. Those parameters were kept constant for each sample, while the deposition time of the Ge core was increased. So, the size of the Ge core increases with the deposition time, while the thicknesses of the shells depend on the sizes of the Ge cores. Each series consisted of four samples. In the first series, there were three samples and one control sample without a shell, with only the core in the matrix. Furthermore, there were three samples in the second series and one control sample without any core and shell and with only Si_3_N_4_ layers in the matrix. The samples were named by the core (Ge) followed by the corresponding core thickness value, and the shell structure (letter A for Al and letter S for Si_3_N_4_). The sample names and sputtering conditions can be found in Table 1.

We used the grazing incidence small-angle X-ray scattering (GISAXS), grazing incidence wide-angle X-ray scattering (GIWAXS), and the Raman method for the structural analysis of the films. GISAXS and GIWAXS measurements were performed at the SAXS beamline of the synchrotron Elettra (Trieste, Italy). A photon energy of 8 keV, a 2D Pilatus3 1M detector (GISAXS), and a 2D Pilatus 100k detector (GIWAXS) were used simultaneously. The grazing incidence angle was slightly above the critical angle. The measured GISAXS maps were analysed using the paracrystal model described in detail in Ref. [27], where the main features of the GISAXS technique are thematised as well. Some more examples and a detailed background of the GISAXS technique are published in Ref. [28]. GIWAXS corresponds to the standard X-ray diffraction measurement under the grazing incidence angle that ensures surface sensitivity.

For the Raman spectroscopy measurements, an Ar ion laser from DILOR Z-24 with a triple monochromator with an excitation line of 532 nm, and power of 50 mW was used. The optical properties of the prepared films were inspected using Ocean Optics equipment comprising a deuterium–halogen light source (DH-2000-BAL), a UV/VIS detector (HR 4000), and SpectraSuite software. Lastly, the electrical properties were investigated using a PTS-2-QE System from Sciencetech in the spectral range from 320 nm to 1200 nm using a bias voltage of 5 V. For this measurement, we deposited one contact over the film, and one on the bottom of the substrate. The first one was a transparent ITO (indium doped tin oxide) contact, and the second one was an aluminium (p-type dopant) contact. Both contacts were deposited using a magnetron sputtering deposition.

## 3. Results

### 3.1. Structural Properties

**Figure 1:**
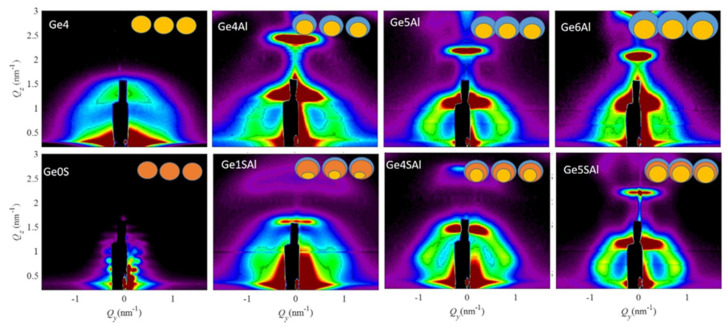
GISAXS maps of all the studied samples. The insets show schematic images of different QD structures that appear in the investigated films. The Ge core is indicated by yellow colour, the Al shell by blue colour, and the Si_3_N_4_ shell by orange colour.

A detailed structural analysis of the prepared films was performed using the GISAXS technique. This method has many interesting features for the thin film’s structure analysis. The method includes many nanoparticles in the measurements, and it is strongly sensitive to the core–shell QDs structure as well as QDs arrangement properties. The method was used to determine the particle shape, average size, ordering properties, and their statistical distribution [17,27,28]. GISAXS maps of all the investigated films are shown in Figure 1.

All GISAXS maps, except the one from the control Ge0SAl sample, show a peak arrangement characteristic for the formation of a 3D lattice of QDs with a BCT arrangement [29]. The ordering quality, i.e., the deviation of the QD positions from the ideal ordering in a BCT lattice, is the best for the films Ge4Al, Ge4SAl, Ge5Al, and Ge5SAl. The ordering quality is closely related to the width of the side peaks centred close to 1 nm^−1^ for all films. A more detailed analysis of the maps was performed by means of a numerical analysis using the paracrystal model of the QD arrangement and assuming their core/shell structure. That model assumes that the QDs are ordered in a 3D BCT lattice with the basis vectors ***a***_1_–***a***_3_, and with the deviations from the ideal positions described by a set of parameters σ_x_–σ_z_. A detailed description of the model is given in the Refs. [27,28].

The shape of the QDs is assumed to be spheroidal with the core radius denoted by *R*_core_, and the total radius *R*_tot_ is equal to the sum of *R*_core_ and the thicknesses of the Al shell (*d*_Al_) and Si_3_N_4_ shell (*d*_Si3N4_), in dependence to the sample. The factor that shows the elongation of the QD in reference to the sphere is denoted as *f*_shape_. It is assumed that the core is placed at the bottom of the shell, as shown in the insets of Figure 1. Thus, the centre of the core is shifted from the centre of the shell toward the substrate by the shell thickness. The shell is asymmetric, i.e., it is similar to a cap around the upper part of a QD. That is realistic because the Ge core is formed first during the deposition and it is covered from the upper side by the shell material [19]. Therefore, we denote the thickness of the shell *d*_Al_ as the difference of *R*_tot_ and *R*_core_. That thickness corresponds to the maximal shell thickness. The thickness of the Si_3_N_4_ shell is denoted by *d*_Si3N4_. The parameters obtained by a numerical analysis of GISAXS maps are given in Table 2.

The size of the core increases, as was intended with the deposition parameters. Values of the radius standard deviation *σ*_R_ are in the interval [0.2–0.8]. The largest standard deviation is found for the film Ge, having no shell. QDs are not created in the sample Ge0SAl, only the multilayer structure is visible. That property is used to make continuous shells around Ge cores that are formed first in the multilayer deposition sequence. 

**Figure 2:**
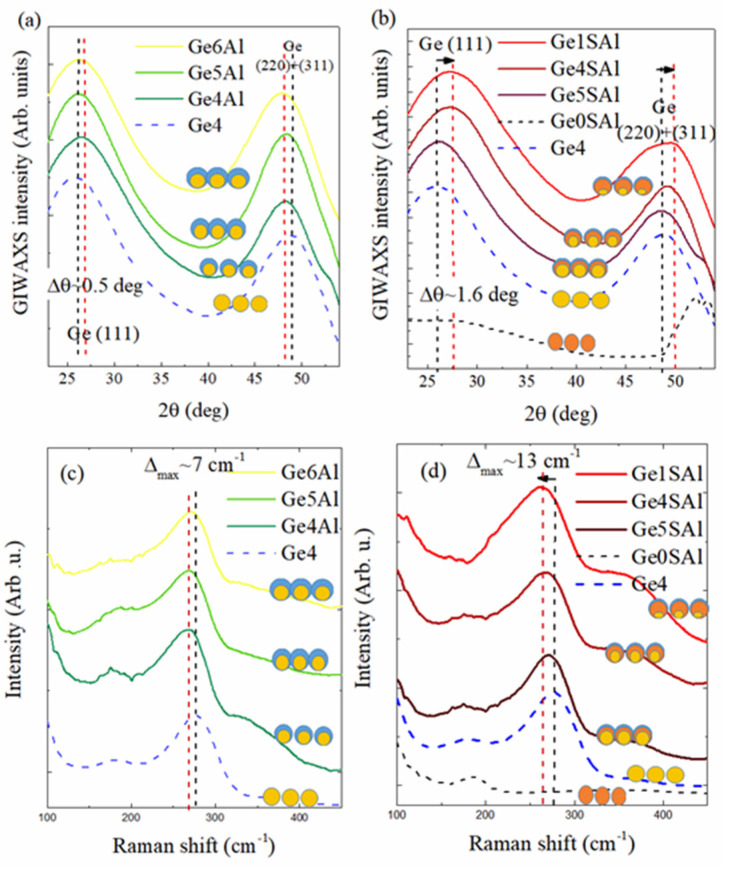
(**a**,**b**) GIWAXS patterns of the investigated films. The black dashed line indicates the positions of the Ge (111) and Ge (220 + 311) peaks for the material without added shells (Ge4), while the red dashed line stands for the maximally shifted peak positions of the same Ge–related reflections. (**c**,**d**) Raman spectra of the same films. The arrows indicate the direction of the peak shift. The symbols show schematic images of different QD structures that appear in the investigated films.

The crystalline structure of the materials was determined using the GIWAXS and Raman methods, as shown in Figure 2. The most significant peaks in both types of the measurements are characteristic of amorphous Ge or small Ge crystallites. The GIWAXS measurements (Figure 2a,b) show two broad peaks around 27 deg and 50 deg, corresponding to the Ge (111) and Ge (200) + Ge (311) peaks, respectively. From the width of the Ge (111) peak the mean size of the Ge nano-crystallites was estimated to be around 1.2 ± 0.3 nm. A weak and broad Si substrate-related (311) peak is visible at 56 deg. Raman data show the Ge-related band close to 275 cm^−1^, which is also characteristic of amorphous Ge or very small Ge crystals. The peaks show widening with the reduction in the Ge core size that indicates a deviation of Ge-Ge bonds in tetrahedrons from the dihedral angle. No Al or Si_3_N_4_ peaks are visible, which is reasonable because of the very small thicknesses of the shells. The alumina matrix is fully amorphous, in accordance with the previous investigations on similar systems [29].

Interestingly, all materials with the shell show a shift of the peaks; a small shift for the first series with the Al shell (Figure 2a–c) and a big shift for the second series with the Si_3_N_4_ shell (Figure 2b–d). This indicates a reduction in the out-of-plane lattice constant, i.e., an increase in the in-plane lattice constant due to the biaxial tensile stress [30]. The peaks in the GIWAXS measurements (Figure 2a,b) shift towards higher angles, while the Raman peaks shift toward smaller values. Both shifts show the existence of tensile strain in the Ge QDs. It seems that the Si_3_N_4_ shell produces a significant strain effect on the Ge QDs. A similar effect was observed in Ref. [21], only the layered structure was used there. 

The shift depends on the Ge core size, and it is larger for smaller Ge core sizes. The largest shift is thus observed for the Ge1SAl sample with the smallest Ge core (1.6 deg for the Ge (111) peak and 13 cm^- 1^ for the Ge-related Raman band). Using the procedure given in Ref. [21], we found a strain of about 3% for Ge1SAl, 1.7% for Ge1Sal, and 1.1% for Ge5SAl. Similar values were obtained using the shift of the Ge (111) peak from the GIWAXS measurements.

The first series, with the Al shell, also shows the shift, but it is much smaller. A maximal shift for the GIWAXS peak of 0.5 deg is observed for this series and a 7 cm^−1^ shift of the Raman peak. The shift is largest for the smallest Ge core size, similarly to the effect of the Si_3_N_4_ shell, but with a smaller amount (maximal strain is 0.9%).

### 3.2. Optical Properties

**Figure 3:**
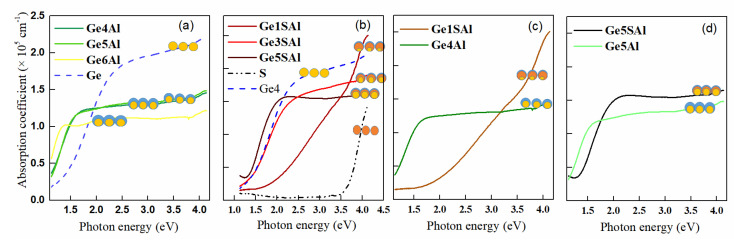
(**a**,**b**) Absorption coefficient as a function of the photon energy. Comparison of absorption coefficients of (**c**) Ge4Al and Ge4SAl films, and (**d**) of Ge5Al and Ge5SAl, differing only by the Si_3_N_4_ shell. The symbols show schematic images of different QD structures that appear in the investigated films.

The optical properties of the investigated films are demonstrated in Figure 3. For these measurements, thin films were deposited on a glass substrate. The spectral transmittance (T) and reflectance (R) of the samples in the spectral wavelength range of 350–1050 nm were measured. From these values and the film thicknesses the absorption coefficient (α) was calculated. Since the Al_2_O_3_ matrix is transparent in the considered spectral range, all absorption effects can be ascribed merely to the QDs and their structure. The measurements show a strong dependence on the deposition conditions.

First, we shall consider the absorption coefficients of the samples with the Al shell only (Figure 3a). The absorption curve of the pure Ge film is relatively broad and increases towards higher energies. The control sample absorbs primarily in the VIS part of the spectrum. The absorption curves of all samples with the Al shell have very similar shapes. The Ge4Al sample has higher absorption in the IR and VIS regions. These three samples show much stronger absorption at energies below 2 eV than the sample without the Al shell. Therefore, this suggests a shift of the absorption edge toward a lower energy, i.e., a shrinkage of the bandgap [22]. This bandgap shrinkage causes a shift in the optical absorption edge from the wavelength of 1.55 µm to ~1.61 µm. The red shift is important to expand the detection wavelength range of the photodiodes [30]. Another important property is that this could reduce the reverse dark current, which is large in Ge photodiodes [22]. The investigated samples show metal-like behaviour with a peak in the IR. 

On the other hand, the curves of the samples with an additional Si_3_N_4_ shell differ significantly (Figure 3b). The control Ge0SAl sample has very low absorption. The Ge4SAl sample has much stronger absorption through all spectral ranges than the other films. The Ge5SAl has weaker absorption, very similar to the two samples from the first series Ge4Al and Ge5Al. The Ge5SAl sample has the biggest amount of Ge, which leads to the formation of larger QDs. That allows a better absorption process, which is in agreement with the red shift toward a bigger wavelength. The Ge1SAl sample has no visible peaks. In the case of this series of the samples, there are two effects that influence the shift of the peaks. The bandgap increases, so there is a shift toward bigger energies (Ge1SAl). Moreover, the bandgap shrinks, so there is a shift toward lower energies (Ge5SAl). The investigated samples show semiconductor-like behaviour with a peak in the VIS. 

The comparison of the absorption coefficients for two films differing only by the presence of the Si_3_N_4_ shell is shown in Figure 3c,d. A relatively big shift towards higher energies and also higher absorption is observed for the films with the Si_3_N_4_ shell. Both shifts are larger for the films with a smaller Ge core (Ge4Al and Ge4SAl). Thus, the structure of these core/shell QDs and their surroundings cause an enhanced absorption. This supports the idea that the presence of an additional shell may advance photon absorption and solar energy conversion.

**Figure 4:**
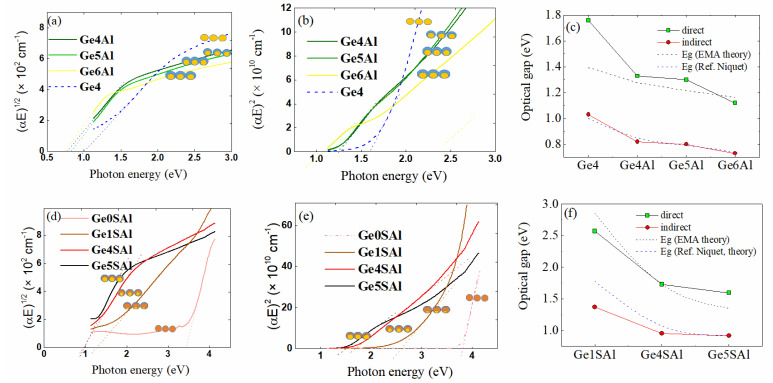
Optical gap determination. Tauc plot for the determination of indirect and direct bandgaps of the prepared materials. (**a**–**c**) Series with the Al shell, (**d**–**f**) series with the additional Si_3_N_4_ shell. The dashed lines indicate the bandgap values in Ge QDs caused by confinement effects, calculated using formulas given in Refs. [32,33]. The symbols show schematic images of different QD structures that appear in the investigated films.

We used the absorption spectra shown in Figure 3 to estimate the optical band gap for the indirect and direct allowed transition, using the procedure described in Ref. [31]. The optical gap determination is shown in Figure 4. The data for the films with the Al shell only are shown in Figure 4a–c, while the results for the films with the additional Si_3_N_4_ shell are shown in Figure 4d–f. Both types of the plot (square root and square of the absorption coefficient times energy) show a linear region, so we used them to estimate the Ge bandgaps, as well as the fitting to the Boltzman function mentioned in Ref. [31]. The obtained values were very similar, and they are summarised in Table 3. The results of the estimation are shown in Figure 4c,f for the Al and Si_3_N_4_/Al shells, respectively. The same figures contain the plot of theoretical estimations of the Ge bandgaps caused by confinement effects. The size of the Ge QDs for the simulations was taken to be the same as the size of the Ge cores in the corresponding samples. The curves were determined according to Ref. [32] (theoretical model given there) and agree quite well with the values of the indirect bandgaps, especially for the films with the Al shell only. We emphasise here that QDs, in our case, are covered by an Al shell, which prevents Ge oxidation—a problem that significantly alters the material’s optical properties. In addition, the QDs in our case are fully isolated from each other (please see the Ref. [19]), so the confinement effect is fully efficient. The value for the sample Ge4, with pure Ge QDs, is slightly larger, while the value for the other samples is slightly smaller. The observed smaller values could be in accordance with the small tensile strain present in the films with the Al shell. In the same curves for the materials containing an additional Si_3_N_4_ shell, the experimental values are significantly lower than the theoretical ones, especially for the ones with the large strain (Ge1SAl and Ge4SAl). This fact is in accordance with the theoretical calculations from Ref. [21], showing that tensile strain causes a decrease in Ge bandgaps.

### 3.3. Optoelectronic Properties

**Figure 5:**
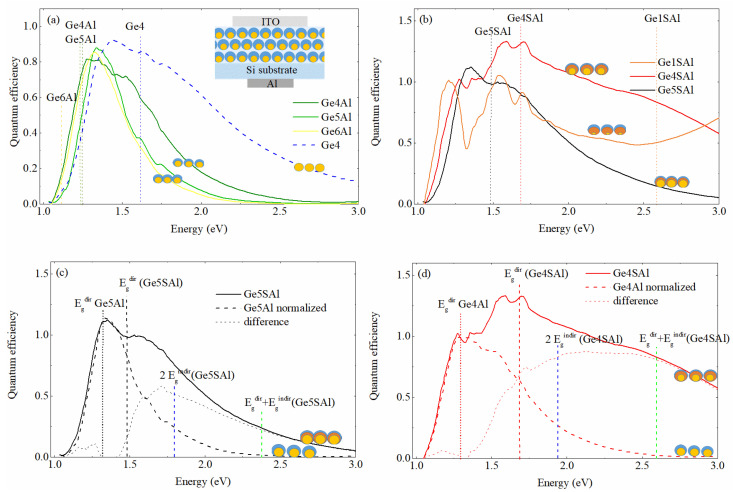
(**a**) Quantum efficiency of the first series of samples normalised to the same maximum intensity. The inset shows a schematic image of the PV device used for the measurements. (**b**) Quantum efficiency of the second series of samples. (**c**,**d**) Quantum efficiency of films Ge5Al, Ge5Sal and Ge4Al, and Ge4SAl with and without the additional Si_3_N_4_ shell. The positions of the direct bandgap and some combinations of the direct and indirect bandgap values are indicated by dashed vertical lines. The symbols show schematic images of different QD structures that appear in the investigated films.

The quantum efficiency (QE) of the investigated materials is shown in Figure 5. The QE curves determined from the Al shell- and an additional Si_3_N_4_ shell-based materials are shown in Figure 5a,b, respectively. The scheme of the geometry for the measurement is given in the inset of Figure 5a. The QE curves represent the number of extracted electron hole pairs per one incident photon. 

The samples of the first series (Figure 5a) show high values of QE in the red region (1.6–2 eV) and especially high values in the IR region (1.1–1.6 eV). The QE curves of the materials with the Al shell (Ge4Al–Ge6Al) are shifting towards lower energy in comparison with the pure Ge (Ge4). This property extends the detection range toward the longer wavelength region. This is in agreement with the optical measurements, and we could conclude that tensile strain induces a small shrinkage of the bandgap [22]. Interestingly, the positions of the QE curves maxima (indicated by dashed lines) agree well with the position of the direct bandgap found in the optical measurements (See Figure 4c) for the films with the Al shell. The QE peak positions are at slightly larger values, which is what is expected. The same is not valid for the pure Ge containing film. 

The QE spectra of the films with an additional Si_3_N_4_ shell show significantly different QE properties, as visible in Figure 5b. The curves are much broader toward the higher energies and also higher in the absolute value. For these curves, the position of the second (and also the highest) peak agrees well with the direct bandgap value determined from the optical measurements. In addition, the maximal QE value of the Ge4Sal sample is larger than 1. This is probably the consequence of the multiple exciton generation effect that is already observed in Ge QDs [1,14]. To resolve better the effects of the Si_3_N_4_ shell addition on the quantum efficiency of the materials, we plotted together the QE spectra of films differing only by the Si_3_N_4_ shell in Figure 5c,d. Interestingly, the shape of the QE curves is almost the same at the lower energies as well as the position of the first peak, while they significantly differ for the higher energies. The difference of these spectra is also plotted in Figure 5c,d. It seems that the presence of the Si_3_N_4_ shell induces the activation of the direct bandgap and probably also some combinations of the direct and indirect bandgap transitions, as well as causes a big contribution to the quantum efficiencies at higher energies. The effect is well visible in Figure 5c, where an additional peak appears slightly above the value of the direct bandgap of the Ge5SAl containing the Si_3_N_4_ shell. The same behaviour, but much more pronounced, is found for the Ge4SAl and Ge4Al films, shown in Figure 5d. For that material, the QE of the Ge4SAl show 250% more extracted electron–hole pairing than the film without the additional shell, only in the measured region. Moreover, the measurements were carried out only up to 3 eV, and the QE from the Ge4SAl film still has quite a high value at that energy, while the value of the QE for the material without an additional shell is close to zero. Thus, the observed enhancement is surely even greater than the 250% observed in the measured range. This may suggest that the additional shell could be employed to substantially enhance the performance of these thin films at higher energies, which is consistent with Ref. [18]. 

The additional contribution to the QE is quite broad, and the value of the QE is larger than 1 for the Ge4SAl film, indicating that multiple exciton generation occurs, i.e., a single photon generates more than one exciton. Therefore, we believe that indirect transitions also occur, so a single photon generates two excitons at the energies close to the double value of the indirect bandgap, and the combination of direct and indirect gaps, as plotted by the vertical dashed lines in Figure 5d.

## 4. Conclusions

In summary, we investigated thin film material consisting of Ge/Al and Ge/Si_3_N_4_/Al core/shell quantum dots regularly ordered within an alumina matrix. The effects of adding Al and Si_3_N_4_/Al shells, and the effect of the Ge core size on the material’s structural, optical, and electrical properties were explored. Our research shows that the presence of an Al shell has a significant impact on the optical and electrical properties of the material. It causes a shift of the bandgap towards smaller energies and it also prevents Ge oxidation. The presence of a thin Si_3_N_4_ shell has a drastic influence on the quantum efficiency of the material. The QE increases in the absolute value, and shows a radically widened energy range with high response. An enhancement of more than 250% in the quantum efficiency of the material was measured. The observed enhancement is the consequence of the tensile strain in Ge QDs of about 3%, which is induced by the addition of the Si_3_N_4_ shell. Such strain should lead to the transformation of the Ge core to a direct bandgap semiconductor. A peak in the quantum efficiency with the position of the Ge indirect bandgap, which actually causes its widening, was observed. We believe that the tensile strain, induced by the addition of the Si_3_N_4_ shell, causes this activation of the Ge direct bandgap, which is one of the main reasons for the strong enhancement of the quantum efficiency. In addition, the contributions of the indirect bandgap transitions, allowing for the observed multiple exciton generations, were also found to be possible. The generated materials are rather promising with respect to their application in various devices, especially those for solar energy conversion.

## Figures and Tables

**Table 1:** Deposition parameters of the produced thin films (left: first series, right: second series); *t* denotes the deposition time. The deposition time for Al_2_O_3_ in each layer was 200 s for all films.

Sample	Composition	*t*__Ge_ (s)	*t*__Al_ (s)	Sample	Composition	*t*__Ge_ (s)	*t*__Si3N4_ (s)	*t*__Al_ (s)
Ge4	Ge/Al_2_O_3_ × 20	40	40	Ge0SAl	Si_3_N_4_/Al/Al_2_O_3_ × 20	---	40	0
Ge4Al	Al/Ge/Al_2_O_3_ × 20	40	40	Ge1SAl	Ge/Si_3_N_4_/Al/Al_2_O_3_ × 20	10	40	40
Ge5Al	Al/Ge/Al_2_O_3_ × 20	50	40	Ge4SAl	Ge/Si_3_N_4_/Al/Al_2_O_3_ × 20	40	40	40
Ge6Al	Al/Ge/Al_2_O_3_ × 20	60	40	Ge5SAl	Ge/Si_3_N_4_/Al/Al_2_O_3_ × 20	50	40	40

**Table 2:** Parameters of the Ge QD lattices determined from the GISAXS analysis: QDs in-layer separation *a* = |***a***_1_| = |***a***_2_|, multilayer period *c* = |***a***_3z_|, deviations of the QD positions from the ideal ones (*σ*_x_, *σ*_y_, and *σ*_z_), core radius *R*_core_, shape factor *f*_shape_, Al shell and Si_3_N_4_ shell thicknesses *d*_Al_ and *d*_Si3N4_, and the standard deviation of their distribution *σ*_R_. The units of all parameters are given in nm.

Sample	*a*	*c*	*σ*_x_	*σ*_y_	*σ*_z_	*R*_Lcore_	*f_shape_*	*d*_Al_	*d*_Si3N4_	*σ*_R_
Ge4	5.8	4.5	5.4	2.5	0.1	1.6	0.97	0	---	0.8
Ge4Al	6.1	5.1	3.1	1.6	0.1	1.7	1.01	1.4	---	0.5
Ge5Al	7.7	5.4	3.2	1.5	0.1	2.0	1.03	1.4	---	0.5
Ge6Al	8.9	6.1	4.3	1.9	0.1	2.2	1.04	1.9	---	0.4
Ge0SAl	---	---	---	---	---	---	---	---	---	---
Ge1SAl	6.8	4.1	3.3	1.5	0.1	1.0	1.03	2.4	2.7	0.2
Ge4SAl	7.2	4.9	2.8	1.4	0.1	1.7	1.04	2.0	3.1	0.5
Ge5SAl	7.5	5.6	2.8	1.7	0.1	2.0	1.1	2.5	3.3	0.6

**Table 3:** Bandgap values calculated from the absorption curves with the corresponding uncertainities. All values are given in eV.

Sample	*E*_g_ ^indirect^	*E*_g_^direct^	Sample	*E*_g_^indirect^	*E*_g_^direct^
Ge4	1.03 ± 0.01	1.767 ± 0.002	Ge0SAl	3.44 ± 0.01	3.877 ± 0.006
Ge4Al	0.82 ± 0.01	1.328 ± 0.007	Ge1SAl	1.37 ± 0.03	2.57 ± 0.03
Ge5Al	0.79 ± 0.03	1.30 ± 0.02	Ge4SAl	0.95 ± 0.01	1.732 ± 0.003
Ge6Al	0.73 ± 0.02	1.12 ± 0.02	Ge5SAl	0.918 ± 0.004	1.593 ± 0.001

## Data Availability

The data available at Mičetić, Maja (2022), “Ge/Al and Ge/Si3N4/Al core/shell quantum dot lattices in alumina: boosting the spectral response by tensile strain”, Mendeley Data, v1, http://dx.doi.org/10.17632/pxms35mv84.1 (accessed on 5 September 2022).
